# Novel online enfacement illusion for investigating self-perception in mental disorders: an experimental study protocol

**DOI:** 10.1186/s40337-024-01026-8

**Published:** 2024-07-05

**Authors:** Jade Portingale, David Butler, Isabel Krug

**Affiliations:** 1 School of Psychological Sciences, The University of Melbourne, Melbourne, VIC 3051 Australia; 2 Faculty of Psychology and Counselling, The Cairnmillar Institute, Melbourne, VIC Australia; 3 Department of Psychology, Counselling and Therapy, LaTrobe University, Melbourne, VIC Australia

**Keywords:** Self-perception, Facial perception, Enfacement illusion, Embodiment illusion, Mental health, Eating disorder, Tactile-reduced, Online

## Abstract

**Background:**

Remote research methods and interventions for mental health disorders have become increasingly important, particularly for conditions like eating disorders (EDs). Embodiment illusions, which induce feelings of ownership over another person?s body or body parts, offer valuable insights into the mechanisms underlying self-perception issues in EDs and potential interventions. However, existing research using these illusions has been limited to face-to-face settings. We illustrate a novel online protocol to induce the enfacement illusion (embodiment illusion principles applied to one’s face) in an ED-based sample.

**Methods:**

Participants complete a 2-hr virtual session with a researcher. First, baseline trait/state ED psychopathology measures and a self-face recognition task occur. Second, participants experience two testing blocks of the enfacement illusion involving synchronously and asynchronously mimicking a pre-recorded actor’s facial expressions. After each block, subjective and objective enfacement illusion measures occur alongside state ED psychopathology reassessment.

**Discussion:**

Successfully inducing enfacement illusions online could provide an affordable, accessible virtual approach to further elucidate the mechanistic role of self-perception disturbances across psychopathologies such as EDs. Moreover, this protocol may represent an innovative, remotely-delivered intervention strategy, as ‘enfacement’ over another face could update negative self-representations in a cost-effective, scalable manner.

## Background

Multisensory bodily (hereafter ‘embodiment’) illusions refer to the illusory experience of perceived ownership, agency, and self-location over another body or body part external to one’s own (e.g., a rubber hand or full virtual body) [1, 2]. These illusions typically arise from synchronising visual input of an external body with tactile stimulation on one's own body and proprioceptive awareness of one’s actual body position and movements [3]. Embodiment illusions have been widely investigated in clinical populations with self-perception disturbances via both real and virtual reality (VR) settings [4], as they offer a means to enhance mechanistic understanding and potentially improve symptomatology. However, these illusions are yet to be conducted outside of a face-to-face environment: limiting more widespread research and potential interventions. Converging fields of research in recent decades have argued that self-perception issues (the inaccurate perception of oneself) are central to the development and maintenance of various mental disorders: for instance, alterations in body representation are linked to the onset and maintenance of eating disorders (EDs) [5], body dysmorphic disorder [6], schizophrenia [7], borderline personality disorder [8], and depression [9]. The current study protocol will present a novel online embodiment illusion paradigm applied to the face, with an example of its application in ED populations.

### Limitations of current face-to-face research and intervention

The coronavirus disease 2019 (COVID-19) pandemic has made evident potential barriers to understanding and treating mental disorders outside of a face-to-face environment [10]. For instance, in a recent survey that investigated the impact of the current pandemic on ED researchers, respondents expressed high concerns about data collection and recruitment, with 20-40% of their current projects being stopped [10]. Moreover, a recent systematic scoping review [11] revealed that up to 61% of studies assessing individuals with various levels of eating pathology reported worsening ED symptoms during the COVID-19 pandemic. The rationale for online interventions for mental health is currently centred on enabling access for those unable to receive evidence-based treatments [12, 13]. For instance, one Australian study reported an average delay of 5.3 years between ED symptom onset and treatment-seeking due to barriers such as costs, geographic constraints, wait times, and stigma [14, 15]. Online interventions enable more flexible, cost-effective delivery reducing wait times over traditional face-to-face approaches [16], with support for their efficacy in treating conditions such as depression [17] and EDs [18].

### ‘Classic’ embodiment illusions involving tactile input

The perception of our body emerges from the combination of a continuous stream of information from the body to the brain [19]. This process entails the integration of various sensory signals (i.e., visual, tactile, proprioceptive, and/or interceptive), termed ‘multisensory integration’, which ultimately combine to form a unified and coherent body representation [19]. However, as the process of multisensory integration is continuous and relatively malleable, distortions of body perception can arise.

**Fig. 1:**
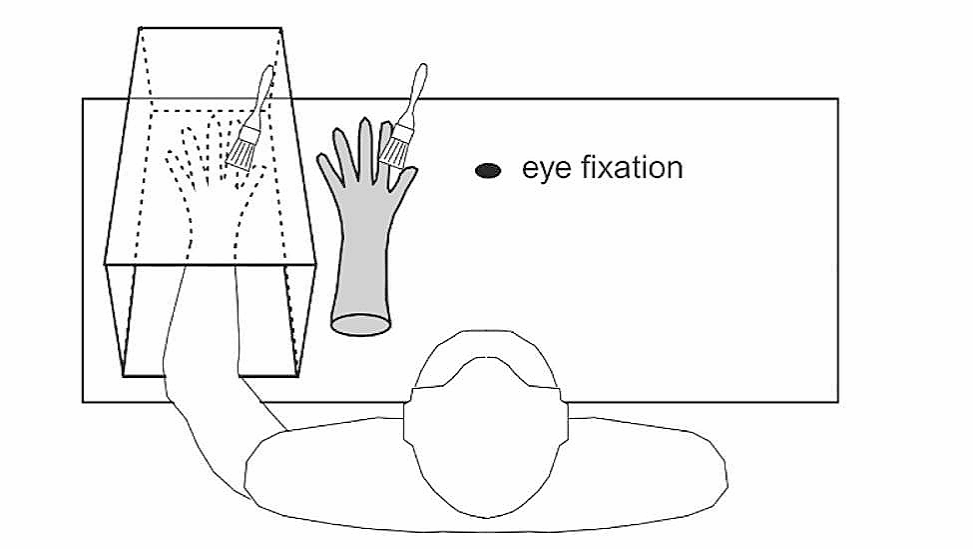
Example depiction of the classic rubber hand illusion procedure with tactile stimulation (i.e., stroking). A participant’s hand is hidden from their view as they observe a rubber hand placed in their view. Both hands are synchronously stroked. Reproduced, with permission, from [82]

This is widely evidenced via embodiment illusions (e.g., the experimentally induced feeling of ownership over a fake/virtual body or body part) which show that it is possible to modulate our internal body representation by inducing multisensory conflicts (e.g., across vision and touch) [2, 4]. These illusions typically involve tactile, visual, and proprioceptive input. In the classic rubber hand illusion (RHI; Fig. 1) [1, 2], synchronous stroking between the rubber hand and the participant’s own (unseen) hand elicits illusory embodiment assessed subjectively via self-report (e.g., perceived *ownership* and/or *agency* over the external body/body part) [20] and objectively such as via changes in body size estimation [21]. Full-body [22] and enfacement illusions [23] employ similar multisensory integration principles (visual-tactile-proprioceptive input, hereafter referred to as ‘tactile’ stimulation) to induce ownership over an entire body or face, respectively, that is external to one’s own. Full-body illusions are often conducted in a VR setting and typically involve the same measures as the RHI. Enfacement illusions are typically induced using a computer screen in a laboratory setting and involve self-report questionnaires (e.g., ownership over the other’s face, appearance similarity) and objective measurement (e.g., self-other discrimination task) [24] (described below).

Individuals with various mental disorders such as EDs [25], schizophrenia [26], and borderline personality disorder [27] demonstrate increased susceptibility to these *tactile* illusions (e.g., a stronger sense of perceived ‘ownership’ over an external body or face typically following synchronous versus asynchronous stimulation), offering insight into the multisensory basis of self-perception issues. Moreover, these illusions can temporarily update distorted bodily representations to improve symptomatology such as reducing body size overestimation post-embodying healthy-weight avatars in individuals with anorexia nervosa [21, 28]. This is grounded in the notion that these illusions–in manipulating the multisensory integration processes underlying self-perception—enable individuals with EDs to temporarily merge their negative self-image with that of another body depicting desirable physical attributes, improving current self-perception [21, 28].

### ‘Tactile-reduced’ embodiment illusions

Recently, tactile-less embodiment via mere synchronous visual-proprioceptive input without tactile stimulation has been supported [29]. For instance, researchers have shown that mere observation of a motionless body part from a first-person perspective induced the RHI in hemiplegic patients [30] and the full-body illusion via VR in non-clinical individuals [31]. Curiously, when compared, embodiment was shown to be stronger following tactile-less relative to tactile stimulation [32], potentially due to the human tendency to weigh visual information over other somatosensory cues [30, 33]. Another tactile-reduced embodiment approach employs visual-motor-proprioceptive synchrony by mimicking bodily movements, successfully inducing full-body illusions within VR [34, 35]. Paralleling tactile approaches, tactile-reduced embodiment illusions show increased susceptibility in disorders such as EDs [31] and schizophrenia [36] and can improve symptoms such as fear of weight gain post-embodying healthy-weight avatars in anorexia nervosa [37].

### Importance of enfacement illusion research

Recent decades have seen the continued emergence of improved, yet cost-affordable VR technologies, with numerous benefits (e.g., enhanced ecological validity by immersing individuals in real-world situations) [38]. Despite this, it is worth considering simpler and more cost-effective embodiment illusion paradigms that do not require excessive technology (e.g., VR headsets), yet maintain ecological validity (e.g., can be conducted in one’s home environment).

The tactile-reduced enfacement illusion, induced through mimicking the facial movements of an actor on a computer screen [24], holds the potential for studying self-perception without VR and external stimuli (e.g., a rubber hand). Furthermore, examining self-face perception via enfacement illusions is crucial for several reasons. The face commonly represents our most distinguishing physical feature [39] and aberrations in face processing such as facial emotion recognition and interpreting expressions are observed in various psychopathologies including alexithymia, autism spectrum disorder, schizophrenia, and mood disorders [40–42]. Moreover, distortions in self-face perception are linked to body dysmorphic disorder and EDs [43, 44], where the face is pivotal in attractiveness judgments [45]. Exploring multisensory mechanisms underlying self-face representation may contribute to an advanced aetiological understanding and interventions for EDs and related conditions. Moreover, as self-face representation is intimately tied to self-awareness [46], this line of research will connect with the broader literature on self-awareness and psychopathology.

A few studies have explored susceptibility to the tactile and tactile-reduced enfacement illusion in relation to mental disorders within laboratory settings. For instance, individuals with borderline personality disorder [47] and schizophrenia [48, 49] have been shown to experience greater susceptibility to tactile enfacement than healthy controls. Regarding intervention, one study demonstrated that experiencing tactile-reduced enfacement with a smiling face via VR (i.e., participants controlled the movements of a virtual face by moving and touching their own face) improved mood in a non-clinical sample as a result of mood migration [50]. This demonstrates the potential utility of tactile and tactile-reduced enfacement methods in understanding and improving mental disorder symptomatology. However, all enfacement illusion research to date has required face-to-face administration in controlled laboratory settings, preventing more widespread application.

### The current study protocol

The present study protocol aims to investigate a novel procedure inducing the tactile-reduced enfacement illusion online via facial expression mimicry, illustrated through application in ED populations. As eating pathology varies from non-clinical and sub-threshold levels in the general community to clinically severe EDs [51], findings from treatment-seeking populations may not fully capture the spectrum of EDs. Hence, we will recruit a sample of women from the community and ED clinics/organisations, encompassing a continuum of ED risk ranging from no history to current, recovered or lifetime ED diagnosis to optimise analyses. The primary aim is to assess whether our online paradigm successfully induces the enfacement illusion by comparing subjective and objective measures of illusion strength across synchronous and asynchronous time points. If so, we will evaluate whether susceptibility to the enfacement illusion differs by ED risk level and whether experiencing the enfacement illusion impacts ED symptomatology and facial and body-related image disturbances. We hypothesise that: (i) our procedure will induce the enfacement illusion (i.e., objective and subjective measures of illusion strength will be stronger/greater post-synchronous relative to asynchronous timing); (ii) individuals with (versus without) ED risk will show increased susceptibility to the enfacement illusion; and, (iii) experiencing the enfacement illusion will improve ED symptomatology and facial and body image disturbances in general, but will be more pronounced in individuals with (versus without) ED risk.

Successfully inducing enfacement online may provide a more affordable, accessible virtual approach enhancing embodiment illusion-based self-perception research across psychopathologies, particularly when face-to-face procedures are unfeasible. Moreover, this protocol may facilitate innovative remotely-delivered interventions leveraging facial embodiment to update maladaptive self-representations. Establishing this paradigm holds important implications for continued self-perception research and intervention accessibility. 

## Method

### Study design

The proposed study is experimental. In a single, 2-hr online session, participants will undergo baseline assessment (T0) of trait/state questionnaires and an objective self-other perception task, followed by two randomised testing phases (T1, T2) involving enfacement illusion induction with subjective and objective enfacement illusion measures and state questionnaires post-illusion.

### Participant recruitment and eligibility criteria

Participants will be recruited from a university and the general community (social media websites, snowballing methods, and personal contacts of the researchers), alongside ED clinics/private practices, ED organisation websites, and ED-related social media pages. Inclusion criteria: (i) women (cis-gender); (ii) aged≥18 years; (iii) Caucasian or Eastern/Southeastern Asian (for model congruency enhancing enfacement/embodiment illusion effects [53–55]); (iv) fluent in English; and (v) access to a smartphone and computer/laptop.

### Stimuli

Eliciting the enfacement illusion involves mimicking the facial expressions of ethnicity-matched models in synchronous/asynchronous videos [26, 50]. Stimuli were created across a pilot phase obtaining ratings of model images to select neutrally-valenced models, and will be generated in the experimental phase involving the creation of face-morphing videos.

#### Pilot phase

##### Face images of models

Images of 10 Caucasian and 10 Eastern/Southeastern Asian female models’ faces (aged≥18 years) were obtained via snowballing. These ethnicities were chosen given that individuals from our target population are primarily Caucasian or Eastern/Southeastern Asian. All images were standardised by having models against a white wall under similar lighting conditions and facing front-on (eye-level) with a neutral expression. Using PhotoScape X (Version 4), as per Tsakiris [56], all images were grey-scaled and given a black background. We then applied an oval frame around the face to remove non-facial attributes (e.g. ears, hair, background). All images were then collated into an online questionnaire via the online survey platform Qualtrics. To minimise bias, an independent community sample of 60 women (30 Caucasian; 30 Eastern/Southeastern Asian) ethnicity-matched to models rated images for facial attractiveness, facial adiposity, likeability, and emotional expression as these factors are known/suggested to bias enfacement illusion effects [57–61]. For example, facial adiposity was assessed using a scale from 1 (*very underweight*) to 7 (*very overweight*). The final 10 models (6 Caucasian and 4 Eastern/Southeastern Asian) were average on all ratings^1^. We combined Eastern/Southeastern Asian faces into a broad ‘Asian’ category, as previous face recognition research has treated them as a single category [62, 63], as do some face databases (e.g., Chicago Face Database) [64].

##### Stimulation video

**Fig. 2:**
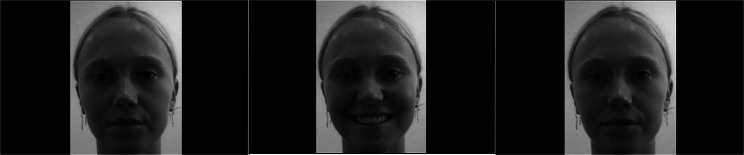
Example depiction of female stimulation video using facial mimicy. Here, the model alternates between a neutral facial expression (left and right) and smiling facial expression (centre) in 10 s iterations for a duration of 150 s

Over a videoconferencing platform (Zoom), the 10 selected models recorded 150 s videos alternating between exaggerated smiles and a neutral expression every 10 s (see Fig. 2). This is consistent with prior protocols [50, 65] and research demonstrating that enfacement is stronger for models with positively-valenced faces [57, 59]. Videos were slightly longer than prior protocols which were typically 120 s [49] to increase the stimulation phase and likelihood of enfacement. Videos were standardised by having models against a white wall under similar lighting conditions and facing front-on (eye-level). Using iMovie, audible sound effects were inserted at the start of each facial expression—smile and then neutral expression—to pace the delivery of each facial expression. The final models received $20 (AUD) e-gift cards as compensation.

#### Experimental phase

##### Face-morphing videos

Participants’ selfie photographs (described below under procedure) will be standardised as per the pilot and flipped horizontally (i.e., mirror-reversed) to match their self-perception (e.g [49]). Using random allocation, each selfie will be morphed with a same-ethnicity (Caucasian or Asian) model’s facial photograph in proportional steps from 100% other to 100% self [56, 66]. Videos will last 100 s, with 100 frames [56]. The other-self direction was chosen given prior research suggesting that only this direction elicits enfacement [20]. The morphed“other” face matches that in the stimulation video.

### Measures

At T0: Eating Attitudes Test- 26 [EAT-26]) [67] assesses ED symptomatology. Using defined cut-off scores on the EAT-26, participants will be stratified into an at-risk ED group (scores ≥ 20) and a not-at-risk ED group (scores < 20).

At T0, and T1 and T2 post-illusion, **state-based** measures:

- Body Satisfaction Scale (BSS) [68]; assesses body satisfaction**/**dissatisfaction with 16 body parts, including a validated 7 items for the body subscale and 7 items for the head/face subscale.
- Shortened 10-item Body Image Concern Inventory (BICI-10 [69]; adapted from Littleton et al. [70]) assesses dysmorphic appearance concern.
- Facial adiposity scale (adopted from Coetzee et al. [71]); assesses perceived facial adiposity (i.e., weight).
- Facial attractiveness scale (adopted from Coetzee et al. [71, 72]; assesses perceived facial attractiveness.
- Self-other discrimination task [24, 56, 58]; participants watch a video of morphed images gradually transitioning from a face that was 100% model (0% self) to 100% self (0% model) and will be required to stop the video (by pressing the space bar with their left-index finger) when the image appears more like the self than the model. Enfacement is believed to occur if participants accept a larger percentage of the model’s facial features as their own (i.e., stop the video later) following synchronous relative to asynchronous interpersonal multisensory stimulation and/or baseline levels of self-other discrimination ability.

At T1, T2 post-illusion: **State-based** enfacement questionnaire [58]; adapted from Tajadura-Jiménez et al. [20], assessing subjective enfacement across self-identification, similarity, and affect.

Post-experiment: Self-reported demographic characteristics (age, gender, current height and weight, main language spoken at home, ethnicity, sexual orientation, highest education completed, and marital status) and ED diagnosis (lifetime, current, or recovered).

### Experimental procedure

**Fig. 3:**
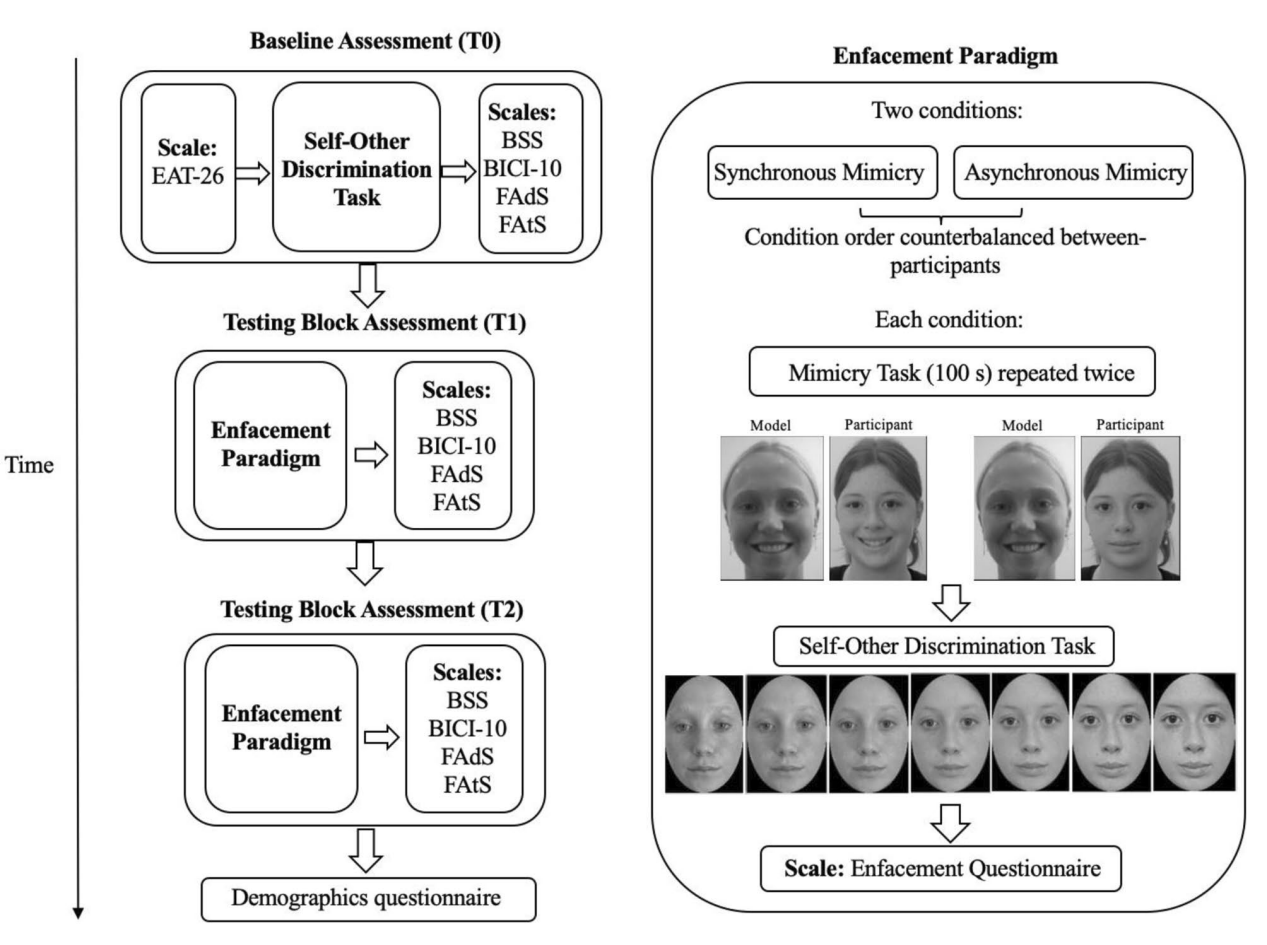
Simplified graphic depiction of the experimental procedure for eating disorder populations containing the three main assessment blocks. EAT-26 = Eating Attitudes Test 26-item; BSS = Body Satisfaction Scale; BICI-10 = Body Image Concern Inventory 10-item short version; FAds = Facial Adiposity Scale; FAts = Facial Attractiveness Scale

See Fig. 3 for a simplified graphic depiction of the experimental procedure. Before the 2 h virtual held over Zoom, participants receive requirements for computer/room set-up (i.e., seated comfortably, approximately 50 cm from the screen, in a quiet, well-lit room) and the selfie. Following consent, participants take the selfie and then complete T0 measures while the researcher prepares the face morphing video using their selfie. Next, participants complete a training protocol involving discriminating their face from an unfamiliar face (none of the assigned models) and completing two face morphing movies (involving well-known celebrities) to ensure comprehension of face identification/self-other discrimination tasks.

**Fig. 4:**
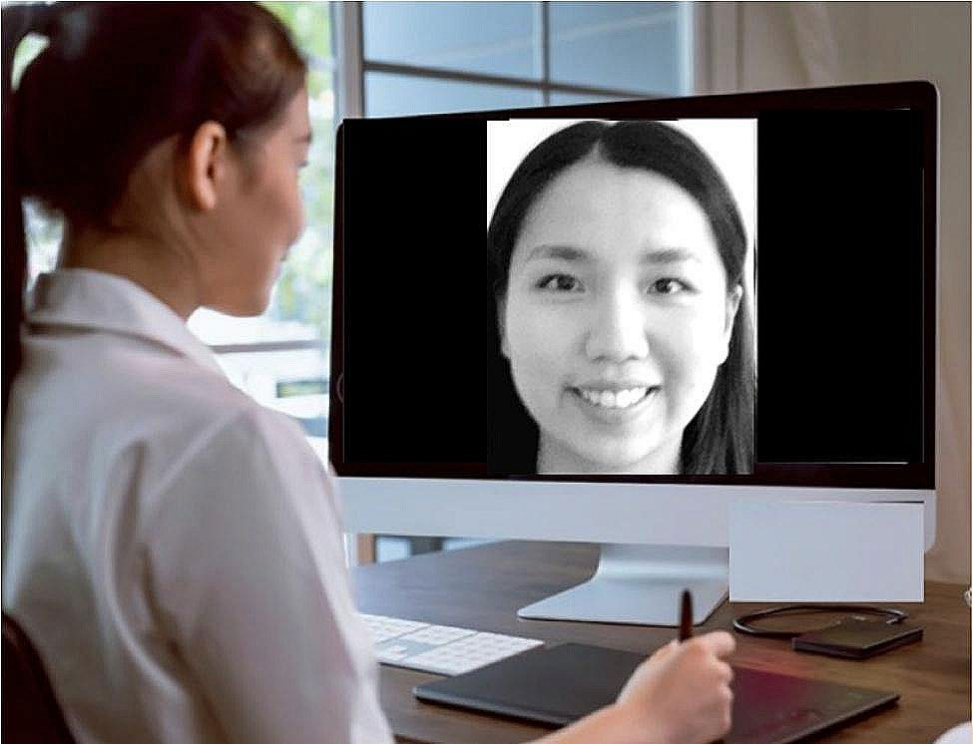
Example depiction of the online enfacement illusion procedure showing the synchronous facial mimicry condition with a smiling expression (i.e., tactile-reduced stimulation)

The main experiment will have two counterbalanced blocks of synchronous/asynchronous tactile-reduced stimulation, each with three phases: (1) Two trials of a mimicry task involving watching the enfacement video and mimicking the model’s facial expressions (see Fig. 4 for a depiction). Participants perform the same facial expression as the model observed in the video (i.e., the participant will smile when the model smiles) and the opposite expression to the model observed in the same video (i.e., the participant smiles when the model displays a neutral expression) during synchronous and asynchronous timing conditions, respectively; (2) The self-other discrimination task and enfacement questionnaire; (3) State-based questionnaires. Post-experiment, participants complete demographic measures and are debriefed and compensated. Standardised procedures are enforced, including mandatory breaks (5 min between each block to prevent carry-over effects and 30 sec between trials of the stimulation video) and strict protocol adherence.

### Statistical analysis

#### Sample size calculation

A target sample ≥ 128 participants (≥ 64 per ED risk group as defined via the EAT-26 [67]) was determined based on an *a-priori* power analysis conducted in G*Power [73] to guarantee a statistical power and statistical level of 95% to detect a medium effect size (*f* = 0.25) [74] in a mixed between-within-subjects ANCOVA assessing ED risk group differences in enfacement susceptibility and improvements across time-points. Analyses will control for important demographic variables (e.g., age). These parameters are consistent with the only previous study assessing the embodiment illusion among a clinical ED population that reported a power analysis [28]^2^.

#### Missing data

Participants with missing data on the EAT-26 will be excluded, as their risk group categorisation is essential. For state-based body image measures, up to 5% missingness will be acceptable given the large target sample size. Minimal missingness on these measures may be imputed if data are missing completely at random or missing at random. Participants with significant missingness will be excluded if data are not missing at random, as imputation could introduce bias.

#### Planned analysis

All analyses will be run using IBM SPSS Statistics (Version 28). Pending that obtained data do not significantly violate ANCOVA assumptions, testing hypotheses will involve mixed between-within-subjects ANCOVAs for each dependent variable. Hypotheses 1–2 will involve a 2 (ED risk versus non-risk) x 3 (Timing; baseline vs. synchronous vs. asynchronous) ANCOVA for objective enfacement (self-other discrimination task); and a 2 (ED risk versus non-risk) x 2 (Timing; synchronous vs. asynchronous) ANCOVA for subjective enfacement (enfacement questionnaire). Covariates will include age, BMI, ethnicity, and alexithymia. Testing hypothesis 3 will involve a series of 2 (ED risk versus non-risk) x 3 (Timing; baseline vs. synchronous vs. asynchronous) ANCOVAs for each body and face-related image disturbance outcome: (i) body dissatisfaction (via the body-related subscale of the BSS); (ii) head/face dissatisfaction (via the head/face-related subscale of the BSS); (iii) dysmorphic concern (via the BICI-10); (iv) facial attractiveness and facial adiposity (via the facial attractiveness and adiposity questionnaires, respectively).

### Ethical issues and dissemination plan

This study has been approved by the local ethics committee (University of Melbourne). Experimental stimuli (stimulation videos and model images) will be made available on Open Science Framework.

## Discussion

Within mental disorders, particularly EDs, bodily misperception remains a critical concern [4], yet research and interventions targeting its underlying mechanisms are limited. The facial region, representing a highly salient aspect of identity, has received even less attention when considering misperception. While technological advancements have increased online ED interventions such as ‘e-therapy’ [12, 13], these methods have not targeted bodily-self-perception. The experimental induction of the enfacement illusion online represents a promising new tool for studying and intervening in facial (and broader bodily) misperception. However, these illusions have not been conducted online outside of face-to-face settings.

The present study will evaluate the effectiveness of a novel online procedure to induce the enfacement illusion via facial mimicry. If effective, this tactile-reduced embodiment illusion method may then shed light on the mechanisms underpinning, and potentially improve, ED symptoms. Its anticipated effectiveness is based on: (1) growing support for experimentally inducing the enfacement illusion within laboratory settings using tactile-reduced stimulation (i.e., facial mimicry) involving mere visual-motor-proprioceptive input [24], and (2) parallels between bodily and self-face perception, with consistent and growing evidence that embodiment illusions offer insights into bodily misperception in EDs and a means to improve ED symptoms (e.g [21, 28]).

This study represents a crucial first step in exploring an innovative approach to comprehending and treating EDs. The proposed online technique could enhance our understanding of self-perception disturbances central to these disorders, whilst highlighting deficits in multisensory integration as a possible underlying factor. Moreover, its potential therapeutic value, when incorporated alongside established interventions like cognitive behavior therapy [75], may offer a promising multidimensional treatment avenue targeting the perceptual, cognitive-affective, and behavioral dimensions of body/face image disturbance. Since tactile-reduced embodiment (e.g [38]) and enfacement illusions (e.g [50]) offer insight into bodily-self-misperception across other mental disorders (e.g., body dysmorphic disorder, schizophrenia, borderline personality disorder, depression), the current method’s broad applicability warrants investigation.

### Strengths, limitations, and future directions

Strengths of the proposed study include the implementation and assessment of a novel online procedure to induce the enfacement illusion, potentially shedding light on mechanisms underpinning bodily (self-face) misperception and improving ED (and other mental health) symptoms. It enables widespread access/availability of research and intervention.

Limitations include potential cognitive fatigue effects from the lengthy experimental procedure which may be particularly detrimental to ED populations [76] and the mixed community and clinical sample assessed based on ED risk potentially yielding smaller effects than a clinical ED sample compared to matched healthy controls. We encourage future researchers to adapt the protocol to clinical populations, ensuring verification of diagnoses. Moreover, future research is needed to determine the best protocol for inducing the enfacement illusion, such as comparing tactile vs. tactile-reduced procedures, examining ideal stimulation duration/number of trials, and investigating facial expressions. For instance, existing research in this area involving face-to-face studies has produced mixed results regarding the latter; e.g., Maister et al. [77] demonstrated that fearful facial expressions yielded a stronger enfacement illusion than other conditions (happiness, disgust, neutral), whilst Beck et al. [78] found no effect of negative facial expressions (fear, anger) relative to a neutral expression on the enfacement illusion. Future researchers should assess differential effects of factors such as stimulation duration/emotional expression and examine the procedure’s applicability across mental disorders and demographics (e.g., gender, ethnicity). Accordingly, recommendations for adjusting the procedure may be made. Furthermore, experiences of plastic surgery and teeth straightening, common in body-image-disturbed populations [79, 80], could potentially influence the results. We encourage future iterations of the current protocol to screen for such experiences in demographic questions to account for their potential influence in the analyses. Lastly, recognising calls in the broader psychological literature for more fine-grained analyses separating major Asian ethnic groups [81], future researchers should attempt to precisely account for ethnic categories. For example, East Asian participants could rate the extent to which their model (East Asian or Southeast Asian) was perceived as ‘typical’ of their ethnic category using a visual analogue scale. This would confirm that participants perceive their assigned model as a member of their ethnic in-group, avoiding potential negative evaluations and enfacement effects due to perceived out-group status [52]. Irrespective of limitations, our research represents a promising first step in this emerging field.

## Data Availability

No datasets were generated or analysed during the current study.

## Abbreviations

ED: Eating disorder
RHI: Rubber hand illusion
VR: Virtual reality

## Footnotes

1 Four of the models were only rated as average in terms of attractiveness and not rated on the other measures as they were adopted from a previous study.

2 Please note that their analysis was based on a simpler ANOVA, thus, producing a smaller target sample of 58 participants, 29 per ED and HC group. Moreover, given the paucity of literature, it is unclear whether the effect size could be large.

## Declarations

Reference Number: 2022-21318-28803-8.

Project Title: Mirror, Mirror on the Wall, That is not what I look like at all: Exploring Self-Perception.

Informed consent to participate will be obtained from all subjects and/or their legal guardian(s).
